# Healthy eating and lifestyle in pregnancy (HELP): a cluster randomised trial to evaluate the effectiveness of a weight management intervention for pregnant women with obesity on weight at 12 months postpartum

**DOI:** 10.1038/s41366-021-00835-0

**Published:** 2021-05-21

**Authors:** Sharon A. Simpson, Elinor Coulman, Dunla Gallagher, Karen Jewell, David Cohen, Robert G. Newcombe, Chao Huang, José Antonio Robles-Zurita, Monica Busse, Eleri Owen-Jones, Donna Duncan, Nefyn Williams, Helen Stanton, Amanda Avery, Emma McIntosh, Rebecca Playle

**Affiliations:** 1 MRC/CSO Social and Public Health Sciences Unit, Institute of Health and Wellbeing, University of Glasgow, Glasgow, UK; 2 Centre for Trials Research, School of Medicine, Cardiff University, Cardiff, UK; 3 Centre for Public Health, School of Medicine, Dentistry and Biomedical Sciences, Queen’s University Belfast, Belfast, UK; 4 Office of the Chief Nursing Officer, Welsh Government, Cardiff, UK; 5 Faculty of Life Sciences and Education, University of South Wales, Newport, UK; 6 Institute of Primary Care and Public Health, School of Medicine, Cardiff University, Cardiff, UK; 7 Hull York Medical School, University of Hull, Hull, UK; 8 Health Economics and Health Technology Assessment, Institute of Health and Wellbeing, University of Glasgow, Glasgow, UK; 9 Abertawe Bro Morgannwg University Health Board, Swansea, UK; 10 Department of Health Services Research, University of Liverpool, Liverpool, UK; 11 School of Biosciences, University of Nottingham, Nottingham, UK

**Keywords:** Lifestyle modification, Weight management

## Abstract

**Objective:**

To assess whether a weight management intervention for pregnant women with obesity was effective in reducing body mass index (BMI) 12 months after giving birth.

**Methods:**

Pragmatic, cluster randomised controlled trial (RCT) with embedded cost-effectiveness analysis. 598 women with a BMI of ≥30 kg/m^2^ (between 12 and 20 weeks gestation) were recruited from 20 secondary care maternity units in England and Wales. BMI at 12 months postpartum was the primary outcome. A range of clinical and behavioural secondary outcomes were examined.

**Interventions:**

Women attending maternity units randomised to intervention were invited to a weekly weight management group, which combined expertise from a commercial weight loss programme with clinical advice from midwives. Both intervention and control participants received usual care and leaflets on diet and physical activity in pregnancy.

**Results:**

Mean (SD) BMI at 12 months postpartum was 36.0 kg/m^2^ (5.2) in the control group, and 37.5 kg/m^2^ (6.7) in the intervention group. After adjustment for baseline BMI, the intervention effect was −0.02 (95% CI −0.04 to 0.01). The intervention group had an improved healthy eating score (3.08, 95% CI 0.16 to 6.00, *p* < 0.04), improved fibre score (3.22, 1.07 to 5.37, *p* < 0.01) and lower levels of risky drinking at 12 months postpartum compared to the control group (OR 0.45, 0.27 to 0.74, *p* < 0.002). The net incremental monetary benefit was not statistically significantly different between arms, although the probability of the intervention being cost-effective was above 60%, at policy-relevant thresholds.

**Conclusions:**

There was no significant difference between groups on the primary outcome of BMI at 12 months. Analyses of secondary outcomes indicated improved healthy eating and lower levels of risky drinking.

Trial registration: Current Controlled Trials ISRCTN25260464.

## Introduction

Around a third to a half of pregnant women in the United States (US), United Kingdom (UK) and Australia are overweight or obese [1–3]. A significant number of women (20–40%) have higher pregnancy weight gain than is recommended [4, 5] which is associated with child obesity [6, 7]. Many women gain weight during pregnancy which they subsequently retain, thus contributing to the development of overweight and obesity [8–10]. Maternal obesity has been linked to increased healthcare costs and risk of complications during pregnancy and birth [11, 12].

A meta-analysis indicated that diet and physical activity interventions reduced gestational weight gain (GWG) [13]. Postpartum lifestyle interventions have also been shown to achieve weight loss [14, 15]. Many studies evaluating interventions for GWG or weight loss postpartum have methodological problems, including issues with randomisation, fidelity, retention, lack of theory and no assessment of cost effectiveness [16–18]. This study, building on a successful feasibility study [19] sought to address some of the shortcomings of previous studies.

The primary study objective was to assess whether a theory-based weight management intervention for pregnant women with obesity, which starts during pregnancy and continues into the postpartum period, was effective in reducing women’s body mass index (BMI) 12 months after giving birth. Secondary objectives were to examine the impact of the intervention on pregnancy weight gain; complications (during pregnancy and postnatally); diet; physical activity; health-related quality of life; mental health; breast feeding and child weight gain.

## Methods

### Trial design

This study was a non-blinded, cluster randomised trial of the healthy eating and lifestyle in pregnancy (HELP) intervention, with a concurrent process and health economic evaluation. This was a pragmatic trial as we recruited patients who would receive the intervention if it were usual care, the intervention was delivered in a usual care setting, there was some flexibility in how the intervention was delivered and it was not possible to blind participants or recruiters. The study took place in England and Wales between February 2011 and June 2014. Twenty maternity units were randomised in a 1:1 ratio between intervention and control arms. Control centre participants received usual National Health Service (NHS) care and were given leaflets on healthy eating and exercise during pregnancy. In addition to usual care and the leaflets, intervention centre participants were also offered the HELP intervention. Participants were followed up at 36 weeks gestation and 6 weeks, 6 months and 12 months postpartum. The study protocol has been published, and a summary of the methods is given below [20].

### Centres and participants

**Fig. 1:**
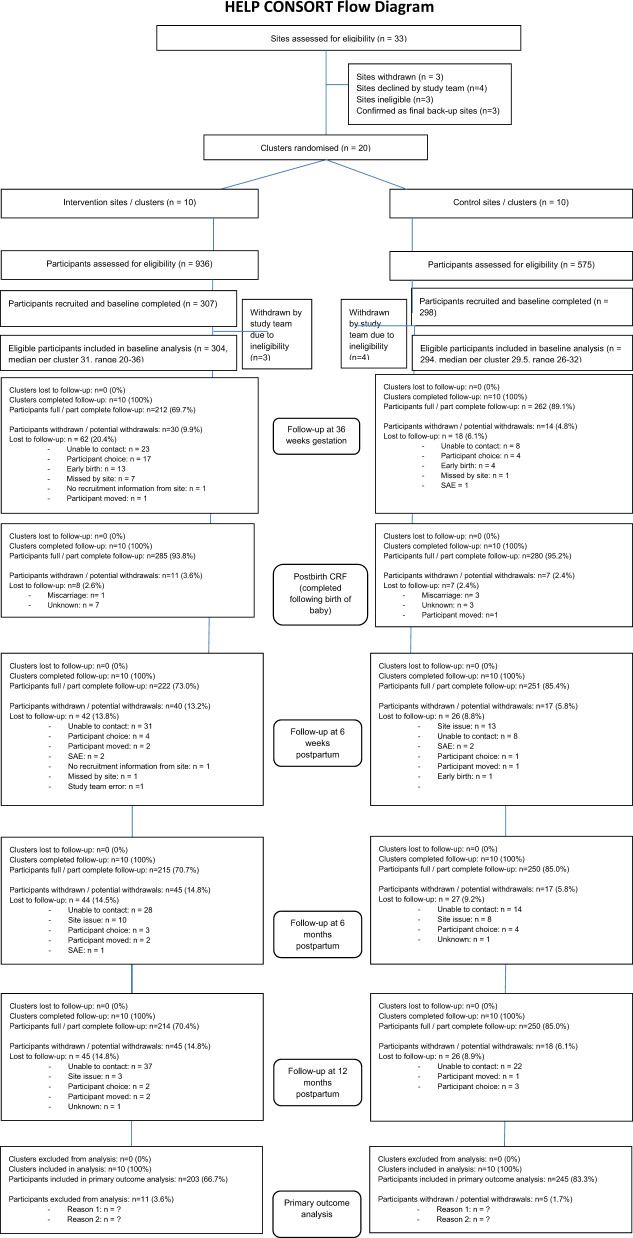
The HELP study CONSORT diagram. HELP CONSORT diagram.

After email contact from the study team, 20 maternity units were confirmed as sites (Fig. 1). Units were chosen to include a variety of demographic profiles including proportion of non-white ethnicities (3–64%; mean 17.8%), proportion of women with BMI > 30 (7–24%; mean 18.5%) and size of unit (births per annum) (1600–8300; mean 4557).

Recruitment and written informed consent occurred after site randomisation. Pregnant women aged 18 years or older with a BMI ≥ 30 kg/m^2^ were approached opportunistically at their earliest antenatal appointment (between 12 and 20 weeks gestation) by NHS midwives or researchers and provided with information about the study. Midwives/researchers approaching and recruiting women were not involved in delivering the intervention.

### Study intervention

The intervention provided support to enhance motivation and equip women with knowledge and skills to enable them to make healthier choices and manage their weight during pregnancy and postpartum [20]. The intervention targeted healthy eating (Slimming World “Extra Easy” programme which follows UK government recommendations) and physical activity (pedometer and walking programme with step targets set and reviewed in group) and included theory-based behaviour change techniques (from Control Theory and Social Cognitive Theory) shown to be efficacious in changing weight-related behaviours [21–24].

Participants attending intervention units were invited to attend free, weekly, 1.5 hour weight management group sessions from recruitment until 6 weeks postpartum (i.e. a maximum of 36 sessions depending when they were recruited and gestation). Groups were held in NHS antenatal clinics and run jointly by a NHS midwife and a Slimming World (SW) consultant. At 6 weeks postpartum women received one voucher for a free SW session at a ‘regular’ community group. The Intervention Midwife also telephoned them at 3 and 6 months postpartum in order to provide longer-term support and discuss weight, healthy eating, physical activity and barriers to success. Further details of the intervention content are published elsewhere [20].

### Primary and secondary outcomes (measured at the patient level)

The primary outcome was maternal BMI at 12 months postpartum. Secondary outcomes were antenatal and birth complications (routine data), pregnancy weight gain, waist circumference and waist-hip ratio, child weight centile, mental health (General Health Questionnaire-12), physical activity (7 Day PAR), diet (DINE), alcohol (AUDIT-C), quality of life (EQ-5D), costs, smoking and breast feeding behaviours (study developed questions) [20].

### Sample size

An individually randomised trial would require 143 women per group to detect a difference in BMI of 1.5 kg/m^2^, based on an assumed SD of 4.5, a two-sided alpha of 5% and 80% power (equivalent to a moderate effect size of 0.333). This difference is in line with weight loss observed in trials including obese women after 1-year follow-up [25]. An intracluster correlation coefficient (ICC) of 0.02 was assumed, which with twenty antenatal units recruiting an average of 20 women gives a variance inflation factor of 1.4 (ICC = 0.02) and a required sample of 400. To allow for loss to follow-up of 30% we planned to recruit 570 women.

### Centre randomisation

The first ten sites were randomised as a block using the optimal allocation method of assignment so that intervention training and recruitment could begin [26, 27]. Randomisation of units was optimally balanced according to geographical location and patient list size, proportion of women with BMI ≥ 30 kg/m^2^ and ethnic mix. This was carried out by a statistician independent of the trial. However, because of delays in setting up the remaining sites, the randomisation method was changed to minimisation (using the balance from the first ten sites) [28, 29]. Subsequent units were randomised as approvals were obtained, maintaining balance for the same four balancing variables using minimisation with the addition of an 80% weighted random component. For these units, the trial statistician based in the Cardiff Centre for Trials Research carried out the randomisation blind to the unit’s identity.

### Statistical analysis

The primary analysis was by intention to treat (ITT), comparing BMI (kg/m^2^) between study groups at 12 months postpartum. For all primary and secondary outcomes, multilevel linear models fitted cluster (maternity unit) and individual effects. BMI data were log-transformed for all regression analyses and baseline BMI as well as variables used to balance the randomisation included as covariates. The intervention effect for BMI was therefore interpreted as the percentage difference between groups. Two-level logistic models were used for categorical outcomes. The impact of individual demographic factors as well as theoretical mediators on the intervention effect was investigated (self-efficacy, social support, intrinsic motivation and self-regulation) [20]. Pre-specified subgroups were examined formally using interaction terms for parity, social class, ethnicity and smoking status. A complier average causal effect (CACE) analysis investigated the effect of intervention group attendance on the primary outcome. The influence of missing data was assessed using multiple imputation under a missing at random assumption. Sensitivity analyses were examined for departures towards missing not at random. All primary and secondary analyses were performed in IBM SPSS Statistics 20 and STATA v13, imputation was performed in STATA v13.

### Economic analysis

A within-trial analysis assessed between group differences in costs against differences in quality-adjusted life years (QALY) derived from the EQ-5D responses from the perspective of the UK NHS and Personal Social Services and following appropriate guidelines [30–33]. Total costs included the intervention, healthcare costs and patients’ out-of-pocket expenses. Reported healthcare resource use and out-of-pocket expenses covered the 3 months prior to each follow-up and were proportionally adjusted to the appropriate time interval. Unit costs for health professional visits and labour costs for intervention midwives were obtained from the Personal Social Services Research Unit [34]. Unit costs for Accident and Emergency department attendance and hospitalization were obtained from NHS reference costs [35]. A multilevel linear model estimated the effect of the HELP intervention on costs at each follow-up, after adjusting for baseline costs and other characteristics. Intervention costs were also calculated for the intervention arm.

EQ5D responses were combined using the UK value set to compute health utilities [36]. A multilevel model estimated utilities at each follow-up, controlling for characteristics and baseline utilities. The area under the curve (AUC) for the within-trial period (17 months) was calculated by adding the quality-adjusted time between follow-up points. A multiple imputation analysis followed methodological guidelines [37]. Missing at random (MAR) was assumed and chained equations were used to predict missing values. Costs and QALYs were estimated for each imputation sample to obtain the distribution of the estimates [38].

The incremental net monetary benefit was computed as INMB = ∆QALY × λ − ∆Cost. Where λ is the threshold for the monetary value of a QALY, ∆Cost is the incremental cost between the HELP intervention and the control, and ∆QALY is the incremental QALY. Two policy-relevant thresholds were used, *λ* = £20,000, £30,000, as per NICE reference case [39]. Probabilistic analysis was performed and the probability of dominance, i.e. the likelihood of an alternative being less costly and more effective, was also computed.

### Code availability and data deposition

The study datasets and code used to generate the results is available for IBM SPSS Statistics v23 and StataCorp LP Stata/IC 13.1 upon request from the author.

### Process evaluation

A mixed methods process evaluation was conducted in line with MRC guidance [39]. Detailed methods are published in our protocol paper. Adherence, contamination and fidelity data are reported here. Intervention group observations to assess fidelity were completed independently by two researchers. Observations were completed twice per site using a structured observation checklist covering key aspects of the intervention [20]. Semi-structured qualitative interviews with participants and focus group with intervention staff were conducted but these will be reported elsewhere.

## Results

### Participant flow

1511 women were screened and 605 were recruited and randomised, with seven subsequently excluded due to non-eligibility. 464/598 (78%) provided primary outcome data at both baseline and 12 months postpartum (Fig. 1). A higher proportion of control participants completed the study (85% versus 70% in intervention arm): this was explored further in the missing data analysis.

### Process evaluation results

When withdrawals are excluded, almost half of the women (49.4%) attended between 26 and 100% of all available sessions (23.4% did not attend any sessions, 27.2% attended fewer than 25%, 14.6% attended 26–50%, 26.4% attended between 51 and 75% and 8.4% attended over 76% sessions). On average participants attended 34% of sessions before birth and 25% of the session after birth. Across the units, the proportion of those never attending varied from 6 to 48%. Agreement between raters assessing fidelity was 84.5% across all sites and all observations. The intervention was generally delivered with good fidelity. Key intervention components including diet, goal setting, self-monitoring and motivation were discussed in 75–100% of observed sessions. The follow-up telephone calls were completed as planned for just over two-thirds of intervention participants, with the other third uncontactable after repeated attempts. However, there was evidence that the physical activity component was poorly implemented, where step targets were only discussed about half the time, with a range of 15.8–76.6% in observed sessions. In addition, few women regularly kept the diaries of physical activity and only 14 provided complete diary data. Qualitative data provided no evidence of contamination across arms.

### Baseline summary data for participants and units

Table 1 shows that at baseline, the recruited sample had a mean BMI 37.2 (kg/m^2^), the BMI was higher in the intervention group. Baseline BMI was included in the primary analysis to adjust for any imbalance. The two arms were reasonably balanced on other baseline characteristics.

**Table 1:** Demographic balance of control and intervention arms at baseline for those recruited to the study.

	Control		Intervention		Overall
	*n*	Mean (SD) or median [IQR] or *n* (%)	*n*	Mean (SD) or median [IQR] or *n* (%)	Mean (SD) or median [IQR] or *n* (%)
BMI (kg/m^2^)	294	36.5 (4.9)	304	37.9 (5.7)	37.2 (5.4)
BMI category	294		304		
30–34.9		128 (43.5%)		118 (38.8%)	246 (41.1%)
>=35		166 (56.5%)		186 (61.2%)	352 (58.9%)
Weight (kg)	294	99.1 (15.2)	304	102.4 (17.3)	100.8 (16.4)
Age (years)	294	28.8 (5.5)	304	29.1 (5.1)	28.9 (5.3)
Gestation (weeks)	293	16.1 (2.4)	304	15.3 (2.7)	15.7 (2.6)
Parity	292	1 [1]	303	1 [2]	1 [2]
Current smoker (n% Yes)	294	39 (13.3%)	304	40 (13.2%)	79 (13.2%)
Cigarettes per day^a^	39	8.8 (6.5)	39	8.7 (4.5)	8.8 (5.6)
Marital status (%)	292		304		
Married/cohabiting		230 (78.8%)		257 (84.5%)	487 (81.7%)
Single/divorced/widowed		62 (21.2%)		47 (15.5%)	109 (18.3%)
Education (% in group)	291		297		
First degree or higher		61 (21.0%)		69 (23.2%)	130 (22.1%)
Stayed in school till 18		95 (32.6%)		80 (26.9%)	175 (29.8%)
Left school at 16		135 (46.4%)		116 (49.9%)	283 (48.1%)
Socio-economic class (% in group)	244		283		
Managerial, professional		93 (38.1%)		148 (46.2%)	209 (42.2%)
Intermediate, small employers		89 (36.5%)		75 (29.9%)	164 (33.1%)
Lower supervisory, technical, semi-routine and routine		62 (25.4%)		60 (23.9%)	122 (24.6%)
Ethnicity (% in group)	292		302		
White		260 (89.0%)		272 (90.0%)	532 (89.5%)
Asian		13 (4.5%)		15 (5.0%)	28 (4.7%)
Black		14 (4.8%)		9 (3.0%)	23 (3.9%)
Mixed/other		5 (1.7%)		6 (2.0%)	11 (1.9%)
Attempting to lose weight before this pregnancy (%yes)	294	237 (80.6%)	302	253 (83.8%)	490 (82.2%)
GHQ	293	114 (38.9%)	302	135 (44.7%)	249 (41.8%)

^a^Based on smokers.

Summary data for clusters (maternity units), confirmed that good balance was achieved overall for the factors used to balance the randomisation: geographic region, ethnic mix, BMI and size of centre. Baseline demographic characteristics by centre indicated balance according to allocation.

Baseline demographic characteristics were similar in women with complete data for the primary outcome at baseline compared to those with missing primary outcome data at final follow-up. However, those with complete data were more likely to be in the managerial and professional socio-economic group (44.9% versus 32.4%) and less likely to be current smokers (10.7% versus 20.7%).

#### Primary and secondary outcomes

Among participants with complete data at baseline and follow-up, the mean (SD) BMI at 12 months postpartum was 36.0 kg/m^2^ (5.2) in the control group, which was lower than in the intervention group BMI (37.5 kg/m^2^ (6.7). This is similar to the baseline difference between arms. In the primary trial analysis (Table 2), the intervention effect was 0.02 (95% CI −0.04 to 0.01). As BMI was log-transformed for regression analysis the intervention effect was interpreted in percentage terms, as a 2% difference in favour of the intervention arm, which was not statistically significant. The ICC was 0.044, indicating 4.4% of the variance in final BMI is accounted for by variations between centre. When additional covariates were added to the final model (women’s age, previous weight loss history variables and mental health) the results were unchanged. Variations in numbers with BMI at baseline and follow up are due to missing covariate data.

**Table 2:** Primary outcome adjusted for baseline BMI and randomisation balance variables.

	Control			Intervention			Adjusted for baseline BMI (log-transformed data), and randomisation variables		
Primary outcome	*n*	Baseline mean (SD)	Follow-up mean (SD)	*n*	Baseline mean (SD)	Follow-up mean (SD)	ICC	Intervention effect and 95% CI	*p*-value
BMI (kg/m^2^)	245	36.2 (4.6)	36.0 (5.2)	203	38.0 (5.9)	37.5 (6.7)	0.044	−0.02 (−0.04 to 0.01)	0.17

Pre-specified subgroup analyses of the primary outcome relating to social class, parity and ethnicity demonstrated no differences in the intervention effect between each subgroup. The smoking status interaction term just reached conventional statistical significance indicating a slightly stronger favourable effect of the intervention in participants who were current smokers at baseline (smoker treatment effect 95% CI: −0.05 (−0.10, 0), *p* = 0.05). These results, as for any exploratory subgroup analyses, should be interpreted with caution.

With regards to secondary outcomes (Table 3), there was no significant difference between groups for any of the weight-related outcomes. However, the proportion reporting attendance at other weight management groups at 12 months follow-up was significantly higher in the intervention group. There were also significant differences between groups on diet and alcohol consumption. The DINE results indicated that the intervention group had an improved healthy eating score and a higher fibre score compared to the control group at 12-month follow-up. The AUDIT-C results indicate the intervention group had significantly lower levels of risky drinking at 12 months postpartum than the control group, even after adjusting for their higher baseline levels.

**Table 3:** Secondary outcomes.

		Control			Intervention			Adjusted for randomisation variables		
Outcome		*n*	Baseline mean (SD)	Follow-up mean (SD)	*n*	Baseline mean (SD)	Follow-up mean (SD)	ICC^a^	Intervention effect and 95% CI	*p*-value
*BMI and weight related outcomes*										
BMI 36 weeks	259		36.3 (4.5)	39.2 (4.5)	208	38.2 (6.1)	40.6 (6.0)	0.008	0.002 (−0.009 to 0.014)^$^	0.977^$^
BMI 6 weeks	247		36.3 (4.6)	36.1 (4.5)	220	38.1 (5.8)	37.5 (5.6)		0.001 (−0.10 to 0.014)^$^	
BMI 6 months	245		36.2 (4.6)	36.3 (5.0)	211	38.2 (6.1)	37.9 (6.4)		0.001 (−0.011 to 0.013)^$^	
Weight at 12 m^b^ (kg)	245		98.4 (15.0)	98.0 (16.3)	203	103.1 (18.2)	101.6 (20.3)	0.043	−0.02 (−0.04, 0.01)	0.17
Waist/hip ratio 12 m	238		n/a	0.86 (0.08)	176	n/a	0.87 (0.07)	0	0.01 (−0.01, 0.04)	0.29
Waist circumference 12 m	239		n/a	106.2 (12.4)	176	n/a	110.3 (15.2)	0.116	4.28 (−0.94, 9.49)	0.11
Proportions:										
Weighing more than weekly	206		75 (36.4%)	85 (41.3%)	173	72 (41.6%)	81 (46.8%)	0.113	*1.14 (0.64, 2.06)	0.65
Exceeding IOM^c^ guidance	259		n/a	90 (34.7%)	208	n/a	66 (31.7%)	0	*0.89 (0.59, 1.36)	0.60
Lost 5% body weight at 6 m	245		n/a	56 (22.9%)	211	n/a	48 (22.7%)	0.250	*1.03 (0.49, 2.14)	0.94
Slimming group attendance^d^	243		170 (72.6%)	75 (32.1%)	194	155 (77.9%)	100 (51.5%)	0.100	*2.13 (1.03, 4.38)	0.04
*Other secondary outcomes*										
AUDIT-C^e,f^		242	23 (9.5%)	107 (44.2%)	202	39 (19.3%)	66 (32.7%)	0.017	*0.45 (0.27, 0.74)	0.002
DINE (HE)^g^		248	2.4 (13.7)	3.6 (13.1)	207	1.9 (13.5)	5.9 (13.8)	0.025	3.08 (0.16, 6.00)	0.04
DINE (FIBRE)		248	28.4 (12.1)	25.6 (12.0)	207	29.2 (11.7)	28.8 (11.8)	0.007	3.22 (1.07, 5.37)	0.01
DINE (FAT)		248	26.0 (10.6)	21.9 (8.4)	207	27.2 (10.7)	23.0 (8.0)	0.027	0.44 (−1.42, 2.30)	0.64
DINE (UFAT)		248	8.0 (1.8)	8.0 (2.3)	207	8.1 (2.0)	7.7 (2.1)	0.000	−0.38 (−0.78, 0.03)	0.07
DINE (FV)		248	5.2 (2.6)	4.8 (2.1)	207	4.9 (2.6)	4.9 (2.6)	0.008	0.37 (−0.09, 0.82)	0.12
7 day PAR (log transformed)^h^		243	88.5 (38.0)	76.8 (24.8)	195	81.0 (27.1)	76.6 (29.9)	0.292	−0.01 (−0.17, 0.14)	0.88

^a^Intra-cluster correlation.

^b^Also adjusted for baseline height and weight (log-transformed data).

^c^Institute of Medicine.

^d^Not including the study intervention.

^e^Proportion reporting higher risk drinking.

^f^There was an error in this questionnaire version that meant the validated score could not be calculated. This score is the total sum of the three AUDIT-C questions converted into a binary outcome 0–3 low risk and 4–6 high risk.

^g^Higher score indicates healthier diet for healthy eating, fibre, fruit and vegetables, lower is healthier for FAT and UFAT.

^h^Intervention effect is interpreted as percentage difference.

*Odds ratio.

^$^Treatment by time effect from repeated measures analysis.

Clinical outcomes included birth weight, pregnancy and birth complications. Gestational age at birth (weeks) in the intervention group was slightly lower than in the control group (difference −0.49 (−0.86 to −0.11); *p* = 0.01). The only other significant difference in clinical outcomes was the Apgar scores at 1 min (0.31 (0.10 to 0.97); *p* = 0.01) where the percentage in the normal range was slightly higher in the control group (51.5% vs 48.5%). All other clinical findings were comparable for these two groups.

#### Sensitivity analyses

Analyses excluding women who were pregnant again at 12 months or had recently had a further baby (*n* = 20 women; 11 control and 9 intervention) did not differ from the primary analyses (−0.02 (−0.04 to 0.01); *p* = 0.20). Addition of women who telephone self reported their weight did not alter the intervention effect in comparison with the primary analysis (−0.02 (−0.04 to 0.01); *p* = 0.16).

A complier average causal effect (CACE) analysis explored the effect of intervention group attendance. Analyses used the number of sessions attended by the participants, or a binary variable dichotomised at a cut off of at least seven sessions for intervention ‘dose’ (55.9% attended <7 sessions and 44.1% at least 7 sessions). By using randomisation as the instrumental variable, the efficacy per session (the incremental effects of each session) attended was assessed. The adjusted results indicate that there was a small efficacy per intervention group session effect on BMI in favour of the intervention which was not significant (−0.001 (−0.003 to 0); *p* = 0.11). Moreover, those attending at least seven sessions showed slightly greater loss in BMI but this was not statistically significant in the binary model (−0.024 (−0.053 to 0.005; *p* = 0.11). This analysis was unadjusted for unit effects and is therefore averaged over all units.

Exploratory mediation analyses did not find any significant mediators of the intervention effect and repeated measures analyses did not alter the primary result.

#### Missing data analysis

To further investigate the possible effect of missing data at follow-up, multiple imputation was carried out under the assumption of missing at random (MAR). A sensitivity analysis was then undertaken in order to assess the effects of departures from the MAR assumption. The result from the combined imputed datasets (*N* = 30) did not differ from the complete case analysis (effect and 95% CI −0.02 (−0.04 to 0.01, *p*-value 0.20).

#### Economic analysis

**Fig. 2:**
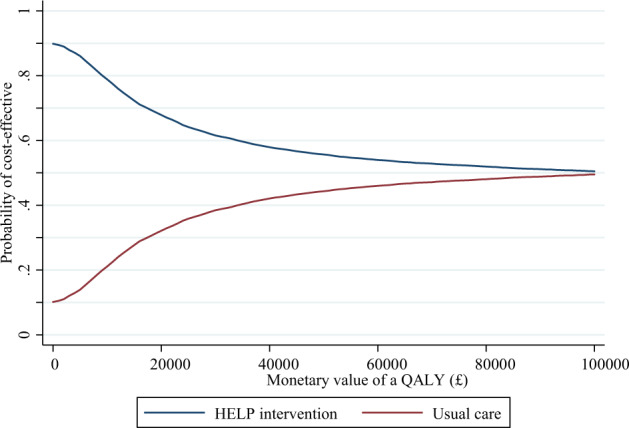
Cost-effectiveness acceptability curve. Multiple imputation.

The percentage of missing values for the intervention arm varied between 0.3%, for utilities at baseline, and 33.9%, for costs at 12 m follow-up. Missing information at each follow up was statistically significantly (all *p*-values lower than 0.02) lower for the control group, with highest missing rate at 22.4%, for costs at 6 weeks postpartum. The cost-effectiveness analysis is shown in Table 4. The total cost per patient (including healthcare, out-of-pocket and intervention costs) was −£404.50 lower for the intervention arm, although that figure is not statistically significant (*p*-value 0.134; CI: −933.1 to 124.1). Also, non-significant QALY losses are estimated for the intervention arm (−0.0024; *p*-value = 0.926; CI: −0.0522, 0.0475). On average, the INMB is higher for the intervention arm at both *λ* = 20,000 and *λ* = 30,000; not statistically significant according to 95% CIs. The probability of dominance is higher for the intervention compared to the control arm. The cost-effectiveness acceptability curve (CEAC) in Fig. 2, shows that the probability of being cost-effective is higher for the intervention group at any threshold below £100,000 per QALY.

**Table 4:** Economic evaluation results: costs per patient (£), QALYs per patient, and incremental analysis.

Alternatives	HELP intervention		Usual care		Incremental	
	Mean	[95% CI^a^]	Mean	[95% CI]	Mean	[95% CI]
Costs (£)	1353.1	[994.7, 1711.5]	1757.6	[1443.5, 2071.7]	−404.5	[−933.1, 124.1]
QALYs^b^	1.2302	[1.1943, 1.2661]	1.2326	[1.2076, 1.2575]	−0.0024	[−0.0522, 0.0475]
INMB^c^ (*λ* = 20,000)					357.3	[−832.6, 1547.2]
INMB (*λ* = 30,000)					333.7	[−1318.7, 1986.1]
Probability more effective (%)	44.9		55.1			
Probability less costly (%)	89.82		10.18			
Probability dominance (%)	41.25		6.53			

Multiple imputation.

^a^95% confidence intervals computed from bootstrapped standard errors.

^b^Quality-adjusted life years.

^c^INMB: incremental net monetary benefit. Dominance refers to the probability of the alternative being both more effective and less costly.

#### Adverse events

A total of 1114 adverse events were reported (502 intervention and 612 control), 245 of these were categorised as serious adverse events (114 intervention and 131 control), none were categorised as related to the intervention.

## Discussion

Although at the 12-month follow-up we observed a lower mean BMI in the intervention arm compared to the control arm (a 2% adjusted difference in BMI), this difference was not statistically significant. The intervention was not effective in reducing BMI at follow-up. The intervention had positive impacts on two secondary outcomes related to diet, where the intervention group reported healthier eating and lower levels of risky drinking at 12 months postpartum. The EQ5D results showed no health utility improvements for the intervention arm and there were no statistically significant lower total costs estimated for the intervention arm.

### Comparison with other studies

Meta-analyses indicate that lifestyle interventions targeting diet and physical activity can impact on GWG and postpartum weight loss [13–15, 40, 41]. Although not statistically significant, the difference between arms in BMI (in the current study) indicated some benefit of the intervention (2% difference) at 12 months. Studies have shown that weight loss of 2–5 kg can reduce the risk of diabetes and cardiovascular risk [41–43]. Although some trials testing lifestyle interventions have shown positive effects on GWG [14, 44–46], like other studies we did not find any statistically significant differences between arms [47, 48]. In the current trial women improved their diet without losing a significant amount of weight, however, this dietary improvement could potentially be beneficial to the development of the foetus. The higher rates of healthy eating found in the intervention arm have been noted in other trials of lifestyle interventions [45, 49], in some cases also without a substantial impact on weight [47]. The economic evaluation findings are consistent with previous studies in that there is no strong evidence that diet-and-physical activity-based interventions in pregnancy are more effective than standard care [50–53]. Total costs per patient (including healthcare, out-of-pocket and intervention costs) in the intervention arm, however, had an 89% probability of being lower than standard care hence future studies should ensure the full spectrum of costs are measured to explore this finding further.

The lack of a significant difference in BMI between arms may be partially explained by the fact that women in both groups were quite intensively followed up and there may have been an impact of this measurement. Also in comparison with other studies, the women in the control group appear to have done well in terms of GWG within IOM guidance. Vinter et al. [46] found that 46.6% of women in the control group exceeded the recommendations, and Dodd et al. [49] found 42% were above these recommendations, which is much higher than the 34.7% in the controls in the present study. This may have made it more difficult to detect differences due to our intervention.

Two other issues may explain the non-significant impact of the intervention on BMI, these are the issues with adherence to the intervention in terms of group attendance, as well as the poor implementation of key aspects of the physical activity component. This may have had an impact on the effectiveness of the intervention, given that diet and physical activity together are most effective for weight loss and this was a key aspect of the intervention [14, 16]. Also since some of the groups had very few women attending at different stages of the study, this may have had a negative impact on social support, a key mechanism of the intervention. Additional support may also be required in the postpartum period, as current evidence in weight loss studies indicates that ongoing support is more likely to lead to effective weight loss [21, 54].

### Strengths and weaknesses

To the authors’ knowledge, this is the first adequately powered RCT including cost-effectiveness, to assess the impact of a lifestyle intervention (for pregnant women with obesity), which starts during pregnancy and continues into the postpartum, on weight at 12 months postpartum. The intervention was designed and piloted following best practice [55] and it was generally well-received both by the healthcare professionals and the women in the study. The study has several strengths including an intervention which was developed based on relevant theory and evidence of effective behaviour change techniques, an objectively measured primary outcome of clinical significance and good retention rates. A large sample of women from across the UK were recruited and although the ICC was higher than we had anticipated for the primary outcome, more participants were recruited than originally planned and retention was higher, so the study was adequately powered. Maternity units across areas of higher deprivation as well as more affluent areas were recruited. The study tested an intervention which had it proved effective could have been rolled out across the NHS, using the same delivery model as current antenatal classes.

There are a number of limitations, one of those was compliance; around a third of the intervention arm did not attend the group or engage with midwife phone calls. This is not unusual for weight loss interventions where attrition varies from 10 to 80% [56]. However, the intervention was quite intensive which likely impacted on engagement. Although we recruited units from across England and Wales with different levels of area deprivation, the sample of women recruited were predominantly white with low representation of different ethnic minorities. This was a pragmatic trial and it was not possible to blind participants or recruiters to centre allocation which could have led to bias in those recruited. There may be some evidence of this as women had higher baseline mean BMI in the intervention group, although we adjusted for baseline BMI. We do not feel that this resulted in systematic bias and allocation concealment was adequate for a cluster design. Drop out was less than expected overall but was higher in the intervention arm than in the control arm. This effect has previously been noted [45]. Imputation was carried out to investigate possible bias and adjust for this, but the conclusions remained unchanged.

### Conclusions

This intervention was not effective in reducing BMI at 12 months postpartum. It did lead to positive impacts on two secondary outcomes; healthy eating and risky drinking. If these reductions in risky drinking and improved diet were sustained they could reduce longer-term risks related to non-communicable diseases. The intervention made a positive contribution but was not enough on its own to produce a sustained effect on BMI. Ongoing support in the postpartum period may be needed to impact on weight loss in the longer term. Pregnancy is a time of significant change in women’s lives and is a potentially important ‘teachable moment’ in which to influence long-term obesity risk. Identifying effective interventions that could provide support to women in the postpartum period and beyond is a vital step in tackling obesity. Benefits to public health could be far-reaching including lower healthcare costs and improvements in women’s physical and psychological health. Future work should explore the factors influencing attendance to try and develop more equitable services that maximize outcomes for all women. In addition, further high-quality trials of interventions are needed which take into account the complex context of weight management in pregnancy and the postpartum. Future interventions will likely need to tackle the problem at multiple levels to be effective.
