# Correction to: Healthy eating and lifestyle in pregnancy (HELP): a cluster randomised trial to evaluate the effectiveness of a weight management intervention for pregnant women with obesity on weight at 12 months postpartum

**DOI:** 10.1038/s41366-021-00976-2

**Published:** 2021-10-18

**Authors:** Sharon A. Simpson, Elinor Coulman, Dunla Gallagher, Karen Jewell, David Cohen, Robert G. Newcombe, Chao Huang, José Antonio Robles-Zurita, Monica Busse, Eleri Owen-Jones, Donna Duncan, Nefyn Williams, Helen Stanton, Amanda Avery, Emma McIntosh, Rebecca Playle

**Affiliations:** 1 MRC/CSO Social and Public Health Sciences Unit, Institute of Health and Wellbeing, University of Glasgow, Glasgow, UK; 2 Centre for Trials Research, School of Medicine, Cardiff University, Cardiff, UK; 3 Centre for Public Health, School of Medicine, Dentistry and Biomedical Sciences, Queen’s University Belfast, Belfast, UK; 4 Office of the Chief Nursing Officer, Welsh Government, Cardiff, UK; 5 Faculty of Life Sciences and Education, University of South Wales, Newport, UK; 6 Institute of Primary Care and Public Health, School of Medicine, Cardiff University, Cardiff, UK; 7 Hull York Medical School, University of Hull, Hull, UK; 8 Health Economics and Health Technology Assessment, Institute of Health and Wellbeing, University of Glasgow, Glasgow, UK; 9 Abertawe Bro Morgannwg University Health Board, Swansea, UK; 10 Department of Health Services Research, University of Liverpool, Liverpool, UK; 11 School of Biosciences, University of Nottingham, Nottingham, UK

**Keywords:** Lifestyle modification, Weight management

Correction to: *International Journal of Obesity* (2021) 45:1728–1739; 10.1038/s41366-021-00835-0, Published online 21 May 2021

The original version of this article unfortunately contained an error in an author name. Jose Antonio Robles should be changed to José Antonio Robles-Zurita. The authors apologize for the error. The original article has been corrected.

